# Dynamic Database Design of Sports Quality Based on Genetic Data Algorithm and Artificial Intelligence

**DOI:** 10.1155/2022/7473109

**Published:** 2022-09-16

**Authors:** Qiang Yue

**Affiliations:** China National Basketball Academy, Shandong Sport University, Rizhao, Shandong 250102, China

## Abstract

According to the traditional data mining method, it is no longer applicable to obtain knowledge from the database, and the knowledge mined in the past must be constantly updated. In the last few years, Internet technology and cloud computing technology have emerged. The emergence of these two technologies has brought about Earth-shaking changes in certain industries. In order to efficiently retrieve and count a large amount of data at a lower cost, big data technology is proposed. Big data technology has played an important role for data with various types, huge quantities, and extremely fast changing speeds. However, big data technology still has some limitations, and researchers still cannot obtain the value of data in a short period of time with low cost and high efficiency. The sports database constructed in this paper can effectively carry out statistics and analysis on the data of sports learning. In the prototype system, log files can be mined, classified, and preprocessed. For the incremental data obtained by preprocessing, incremental data mining can be performed, a classification model can be established, and the database can be updated to provide users with personalized services. Through the method of data survey, the author studied the students' exercise status, and the feedback data show that college students lack the awareness of physical exercise and have no fitness habit. It is necessary to accelerate the reform of college sports and cultivate students' good sports awareness.

## 1. Introduction

As of today, many information processing modes that are only used in the study of biological evolution have been used in computational intelligence methods [1]. After training the data and establishing the connection between the data, many problems have been solved through computational intelligence. In all areas of human society, the combination of computer intelligence and artificial intelligence is very common [2]. The most important part of genetic algorithm in evolutionary computing has been applied to various fields, but users have found that the algorithm has certain problems in its application. It takes a long time to find the optimal solution, and the similarity between individuals is too great and high, unable to search the solution space [3]. When using genetic algorithms, binary coding is generally adopted, but this coding method cannot reflect the influence of genetic information on the growth of organisms. Therefore, the regulatory role of DNA cannot be reflected in subsequent calculations using the calculation model [4]. At the end of the 20th century, researchers developed a new molecular biological calculation method for DNA and biological enzymes [5]. This method uses the principles of biochemical reactions to calculate, which is more cutting-edge. Intelligent computing has attracted the attention of a wide range of researchers as soon as it was proposed. However, the research on intelligent computing has always been carried out in the laboratory [6]. Experiments can only be carried out using biology, and the method cannot be directly applied to the engineering field to solve problems. With the advent of DNA computing, researchers have discovered that intelligent systems based on DNA can better reflect biological genetic information, which is conducive to promoting the development of artificial intelligence [7]. Under this discovery, researchers integrated DNA algorithm into genetic algorithm, imitating biological genetic information, and formed genetic algorithm [8]. Evolutionary algorithms include a variety of algorithms. Different algorithms not only have slight differences but also have common points when calculating. All evolutionary algorithms rely on the idea of biological evolution when solving problems. When conducting research, researchers usually prefer to use genetic algorithms in evolutionary algorithms [9]. The concept of genetic algorithm and self-adjustment came into being at the same time in the middle of the 20th century. At that time, there were special books on the principles of genetic algorithm. After Goldberg completed his monograph, the genetic algorithm was formally formed. It can be applied in various fields and has attracted wide attention from people from all walks of life [10]. Although genetic algorithms that use the idea of population evolution and natural selection to find the optimal solution cannot get the most advantages, they can continue to find more advantages in the process of continuous calculation.

## 2. Related Work

When discussing the calculation process of the genetic algorithm, people usually have the illusion of simple calculation, but in fact, the operation mechanism of genetic algorithm is particularly complicated. According to the author's research, the theoretical basis of genetic algorithm is still developing, and its application is becoming more and more extensive. Genetic algorithm has played an important role in solving complex problems and engineering design and has successfully attracted the attention of researchers. The research results are the following.

Many scholars around the world have put forward specific opinions on the coding problem of the genetic algorithm. In order to make it more convenient to calculate the length of the binary genetic algorithm, experts proposed a unified calculation formula, which solved the problem of extracting length, which is beneficial to computer programming [11]. After considering the two factors of decision variables and interval length, the smallest code length is selected to avoid inefficiency problems and, at the same time, to avoid discrete errors, which greatly improves optimization efficiency. The literature proposed a new genetic algorithm in order to solve the problem of too many parameters in the optimization design [12]. This algorithm is formed on the basis of weighted real number coding and is a development of the weighting idea in binary coding. In the literature, the number of each layer is different when the damper is installed. In order to solve these problems, a new optimization layout method is proposed on the basis of the genetic algorithm of digital sequence coding. Some research puts forward suggestions for improving the genetic algorithm of real number coding [13].

In the research of genetic algorithm, genetic operation is the main content of the research, and its research is to improve the efficiency of calculation. The literature improved the problem of premature convergence in the current genetic algorithm from two aspects: probability and operator [14]. The literature proposes the mutation of the confounding sublist, but the confounding part should not be too much. The literature solves the problem of illegal operation by modifying the program. The literature proposes to allow the probability to be adaptively selected [15]. It pointed out the necessity of the interchange mutation method. The research on the probability of chromosome selection has promoted the progress of genetic algorithms. Because of the advantages of mutation that can preserve the diversity among individuals, researchers have optimized the operation of mutation. Literature mutated individuals according to their strength [16]. It generates new individuals based on location information to search different areas.

## 3. Research on Genetic Data Algorithms and Artificial Intelligence Big Data

### 3.1. Genetic Data Algorithm

**Figure 1:**
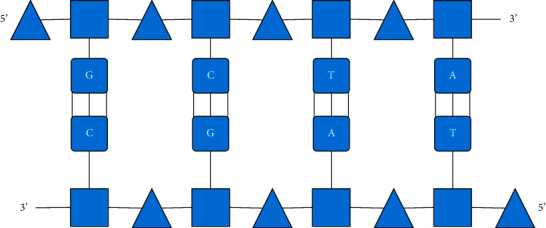
DNA molecule base-pairing principle.

When people have further results in the research of modern molecular biology, it is discovered that genetic information is stored in the DNA of organisms. In order to be able to copy genetic information faster, it is necessary to ensure the specificity of the protein, and molecules play an important role in the calculation process, as shown in Figure 1.

**Figure 2:**
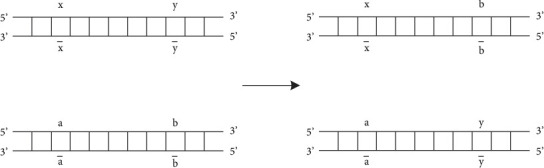
DNA recombination.

According to Figure 1, it can be found that there are two different elements on the skeleton, which play a decisive role in the direction of the chain. The process of exchanging different molecular fragments in DNA is the process of recombination. The specific process is shown in Figure 2.

We use the function to express the base-to-complement base mapping:

(1) $$\begin{array}{c} W\left(A\right)=T,W\left(T\right)=A,W\left(C\right)=G,W\left(G\right)=C\text{.} \end{array}$$

The letters in the function can be mapped one by one:

(2) $$\begin{array}{c} W\left(TTCGC\right)=AAGCG\text{.} \end{array}$$

By manipulating the expression method of DNA genetic change sequence, the sequence is mapped into the problem space. The optimization problem with variable *n* can be expressed as follows:

(3) $$\begin{array}{c} \left\{\begin{array}{c} \min\;f\left(x_{1},x_{2},x_{n}\right)\text{,}\\ x_{i_{\min}}\leq x_{i}\leq x_{i_{\max}},i=1,2,n\text{.} \end{array}\right. \end{array}$$

When decoding, the length and variables of the encoding must be considered, and the decoding model must be described.

First, to convert the subchain of the variable into an integer value, the formula used is as follows:

(4) $$\begin{array}{c} teempx_{i}=\overset{l}{\underset{j=1}{\sum }}bit\left(j\right)\times 4^{i-j}\text{.} \end{array}$$

The value of the subchain can be transformed by formula (5), and the corresponding solution can be obtained:

(5) $$\begin{array}{c} x_{i}=\frac{tempx_{i}}{4^{l}-1}\left(x_{i_{\max}}-x_{i_{\min}}\right)+x_{i_{\min}}\text{.} \end{array}$$

### 3.2. Population of DNA Genetic Algorithm

The steps to improve the DNA algorithm are as follows:

(1) Get the fitness of each individual.
(2) Determine the probability of an individual being inherited to the next generation:
  (6) $$\begin{array}{c} P\left(x_{i}\right)=\frac{f\left(x_{i}\right)}{\overset{N}{\underset{i=1}{\sum }}f\left(x_{i}\right)}\text{.} \end{array}$$
(3) Roulette is obtained by calculating the probability of each individual:
  (7) $$\begin{array}{c} Q\left(x_{i}\right)=\overset{i}{\underset{j=1}{\sum }}P\left(x_{j}\right)\text{.} \end{array}$$
(4) Find the balance between global search and local search. The specific calculation method is as follows:
  (8) $$\begin{array}{c} \left\{\begin{array}{c} P_{ml}=a_{1}+\frac{b_{1}}{1+\exp\;\left[-c\left(g-g_{0}\right)\right]}\text{,}\\ P_{mh}=a_{1}+\frac{b_{1}}{1+\exp\;\left[c\left(g-g_{0}\right)\right]}\text{.} \end{array}\right. \end{array}$$

**Figure 3:**
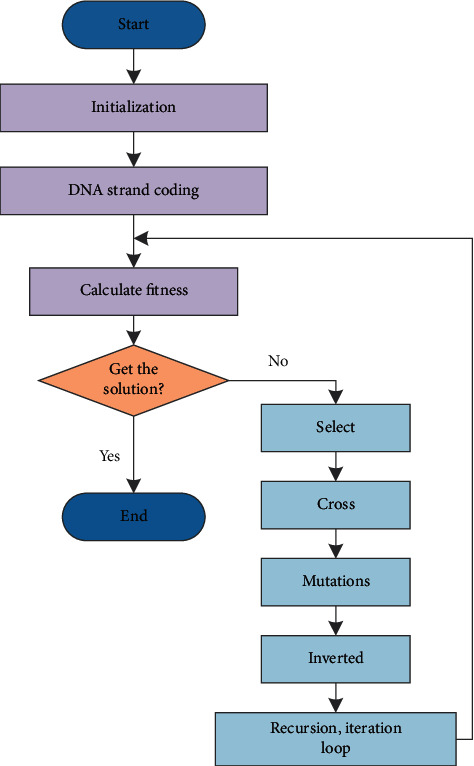
DNA genetic algorithm flowchart.

In the process of promoting population diversification, the structural characteristics of biological DNA molecules are used for inversion operations. In the subsequent calculations, the inversion operation described here is also used many times. The specific evolution flow chart is shown in Figure 3.

The probability of a group generating an individual can be obtained by multiplying multiple probability matrices:

(9) $$\begin{array}{c} P=SCMI\text{.} \end{array}$$

The crossover operator used in the calculation has the following characteristics:

(10) $$\begin{array}{c} \overset{size}{\underset{a=1}{\sum }}\overset{size}{\underset{b=1}{\sum }}C_{ij.ab}=1\text{.} \end{array}$$

After mutation conversion, the probability matrix is as follows:

(11) $$\begin{array}{c} M=\overset{size}{\underset{a=1}{\sum }}m_{ay}\text{.} \end{array}$$

According to the algorithm,

(12) $$\begin{array}{c} m_{ay}={\left(\frac{p_{m}}{3}\right)}^{H\left(a,y\right)}{\left(1-p_{m}\right)}^{L-H\left(a,y\right)}\text{.} \end{array}$$

The inverted conversion probability matrix is as follows:

(13) $$\begin{array}{c} I=\overset{size}{\underset{a=1}{\prod }}{\left(\frac{p_{i}}{3}\right)}^{H\left(a,y\right)}{\left(1-p_{i}\right)}^{L-H\left(a,y\right)}\text{,}\\ \rho ={\left[\min\;\left(\frac{P_{i}}{3},1-P_{i}\right)\right]}^{NL}\text{.} \end{array}$$

### 3.3. Artificial Intelligence Big Data

In the near future, artificial intelligence will have broader application prospects. It can truly realize automation and liberate human hands. Big data is very important in the process of promoting the development of artificial intelligence. There is a large amount of data to provide support for researchers in the research and development of artificial intelligence. Big data has the following characteristics: first, there are a huge amount of big data, and second, there are many types of big data, including data from various industries. Third, big data has high application value. Finally, it has a faster processing speed. In addition to the research value of big data itself, the core idea of research is also changed when applying big data. People study artificial neural networks in the hope that computers can imitate the human brain for thinking in the near future. People have conducted 40 years of research in this field, so the accuracy rate is high. For example, the application of artificial neural network to the monitoring system can accurately identify and calculate noise and propose improvements, which is conducive to improving the efficiency of monitoring.

In the research process, the concept of smart city is proposed by combining the Internet of Things and the Internet. An ideal city model that can be controlled in the real time is simulated by computer technology. To build a smart city, we must rely on a certain information foundation and database and at the same time promote the intelligence of various structures and systems in the city. In the construction of smart cities, big data technology can not only provide urban pollution data but also predict economic development, playing a very important role.

With the development of artificial intelligence, there will be further progress in the field of commercial deployment of speech recognition research. In the research in this field, how to process natural language and make rational use of machine learning has always been the focus of research. In the application of artificial intelligence, natural language processing has made great progress, and related voice products will be commercially deployed in the near future. The artificial intelligence industry and smart cities promote each other, and smart cities not only bring new directions to the development of artificial intelligence but also promote the progress of smart cities and promote the improvement of artificial intelligence technology. With the development of research, rapid progress has been made in the field of service period robots. It can be applied to all aspects of people's lives and has a good market prospect.

## 4. Research on the Dynamic Database of Sports Quality

### 4.1. Sports Quality

In a certain city, 490 students from different schools and different majors were selected, including 250 boys and 240 girls, to investigate the physical fitness of these students. During the research process, questionnaires related to students' physical fitness were set up, and 490 questionnaires were distributed to students. After removing invalid questionnaires, 472 questionnaires were left to check the credibility of the questionnaire content. Analysis of the questionnaire data shows that college students lack awareness of physical exercise. Only 8% of students take more than two hours of physical exercise a day. Students who exercise for less than half an hour or even do not exercise each day account for 51% of the total. These data not only reflect that college students spend less time on physical exercise every day but also show that colleges and universities do not pay enough attention to physical exercise. Among all students participating in sports, ball sports accounted for 37%, running accounted for 25%, gymnastics accounted for 6%, and other sports accounted for 32%. This shows that some students are not interested in sports, and it also reflects the lack of technical guidance for students in sports. According to the data in the questionnaire, the number of people who can reach the pass line in the 1000-meter test accounts for 50% of the total number, and the number of students who fail to pass the sit-ups reaches 60%. From these two data, it can be seen that the students' physical fitness is poor. 60% of people clearly stated that they do not like sports, and 5% of people think that the effect of physical exercise is average. It can be seen that students lack the interest in physical exercise and cannot correctly understand the benefits of physical exercise to the body. Students need to cultivate their interest in sports.

1. *The School Does Not Pay Attention to Physical Education for Students.* Compared with ordinary colleges and universities, the education of higher vocational colleges pays more attention to the cultivation of students' professional knowledge and the practicality of knowledge. In order to promote the development of higher vocational education, higher vocational colleges pay attention to the cultivation of students' professional knowledge when teaching. The teaching goal is mainly to cultivate students' on-the-job ability, cultivate skilled talents, and meet the needs of society for talents. Therefore, in the education of students, the teaching of physical education is ignored, and the training of students' sports quality is not emphasized. In the teaching process, teachers only pay attention to whether the teaching content is completed and whether students can successfully pass the final assessment and did not really improve the physical fitness of students. The content of physical education courses in some higher vocational colleges has not even been implemented, and students focus on free activities during class. When cultivating students, it pays too much attention to students' progress rate and ignores students' health. Colleges and universities hope to cultivate talents that meet the needs of society as soon as possible and regard them as the only goal of education.
2. *The Goal of Physical Education Is Fuzzy.* In the physical education of higher vocational colleges, there is a problem of vague teaching goals. The overall goal of teaching is to enhance the physical quality of students and cultivate their ideological and moral qualities. However, the specific connotation and level are not clear, which seriously affects the setting of teaching tasks, and it is difficult for teachers to choose the correct teaching methods and teaching content. In addition, the teaching plan in the physical education classroom is not clear and is restricted by physical education teaching materials. In the teaching process, teachers are only responsible for teaching and do not pay attention to students' learning. The teaching content is single, the organization form is backward, the performance evaluation method is outdated, and student health is not put first.
3. *Backward Teaching Ideas and Insufficient Sports Talents.* Students especially hope that teachers have high-end professional skills and provide students with a relaxing sports environment. They hope that teachers can be enthusiastic enough to make beautiful movements. But at present, the overall quality of physical education teachers is not high, and the overall academic qualifications of teachers are low, so they cannot meet the needs of students. Under such realistic conditions, the physical education teaching model must be reformed in time. In the teaching process, some teachers are relatively backward in thinking and only pay attention to the teaching of sports knowledge and skills. In the course of class, teaching materials are the center. This teaching method seriously hinders the improvement of students' physical fitness and is not conducive to the development of students, the habit of physical exercise.
4. *Outdated Sports Assessment Standards and Insufficient Sports Equipment.* During the research process, the author found that the content of the assessment of students' physical fitness in colleges and universities is fixed. Due to the existence of scores, students will practice the content of the examination in consideration of credits. Once the assessment is over, the motivation of students to exercise will be greatly reduced. Many students stop the corresponding physical exercise after the assessment is over. This method is not conducive to promoting students to develop the habit of exercise. In some schools, various elective courses of physical education have been developed, and students can choose the courses according to their hobbies. However, under normal circumstances, students' course selection is limited to one semester. Each semester only has more than ten weeks, and there is only one physical education class per week. Sometimes physical education classes will be suspended due to holidays or weather. Because each student's physical fitness is different, and the ability to master new knowledge is also different, there are differences in students' exercise levels, and it is unscientific to use a unified evaluation standard to assess students. In the actual investigation, it was found that, for the same physical education class and the same physical content, some students need to spend more time studying to barely pass, while some students only need to exercise in class or even before the examination. Learning can achieve good. There may even be some students. Because of their physical fitness, it is difficult to pass even if they exercise hard, so the enthusiasm of the students will be greatly reduced. In addition, some colleges and universities have inadequate sports facilities. The sports equipment is relatively old, and the lack of hardware facilities has seriously hindered students from playing sports.

### 4.2. Research on Dynamic Databases and Related Technologies

When people visit a website, they leave access information, which is recorded in log files. Through log analysis, users with similar behaviors can be found. Analyzing the access status of the page can greatly shorten the conceptual difference between the designer and the visitor. In this article, the author analyzes the data source and data processing process and proposes a method to solve the log incremental mining.

The server records the visitor's browsing behavior in the form of a log. The specific visit information includes user name, IP, and time. The storage of this information has a standard form, so it is very convenient to extract. The main problem encountered in this process is how to group user requests. Generally, there are two methods that can be used, one is to track user requests, and the other is to use detectors to capture user behavior. However, the first method requires the user's consent, but even with the user's consent, the accuracy of the identification information cannot be guaranteed. The second method has certain difficulties in data transmission and is prone to data leakage problems.

In order to avoid problems in the identification of things on the client side, a remote agent can be used. In this way, although more detailed user information can be obtained, this process requires users to provide privacy. Compared with receiving information from the server, receiving information from the client can solve the problem of thing identification, but it also generates higher costs. There are many proxy servers on the proxy side, which can reduce the time of network transmission load. There is no more difference between the data collected from the agent layer and the data collected from the service layer except for the difference in quantity.

There are certain problems with the data in the real-world database. For example, the data may be incomplete due to the deletion of the data when inputting the data. If the equipment fails when collecting the data, the data obtained may be noisy. If there is a problem with the data source, the result of the data must be inaccurate. The existence of these problems affects the effectiveness of the algorithm. Therefore, it is necessary to preprocess the data, remove the noise in the data, and standardize the data when mining the data, so as to improve the accuracy of the data. Preprocessing log files can delete irrelevant data generated in the data mining process.

Different logs contain different user information, and the relevant logs should be consulted according to the needs of users. Several simple access logs are recorded in Table 1.

**Figure 4:**
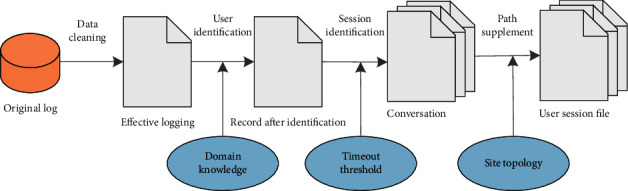
Web log file preprocessing process.

Analyzing the log in Table 1, only when the return code is 200, it is a successful access. The steps of preprocessing the log file are recorded in Figure 4.

Data cleaning is to remove irrelevant data in the log file. Some automatic tracking software may send the wrong user agent. In this case, it is necessary to separate users from things. When cleaning up, we check the content and URL of the image page.

**Figure 5:**
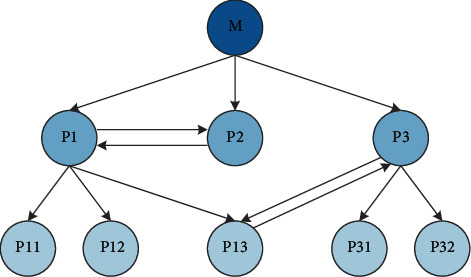
A simple WEB topology.

User identification will be affected by the local cache and firewall. If the user returns to the previous page while constantly clicking the page, the behavior of returning to the page will not appear in the log. The proxy server can provide intermediate cache to users, so in the same period of time, the same page can be accessed by multiple users, and the complexity of the log is greatly improved. The purpose of a firewall is to protect user information, but it also prevents logging of IP addresses, which makes it difficult to identify users. Users want to be able to get better service when browsing the website, but they do not want their behavior to be tracked. For websites, it is hoped that they can understand customers through tracking and provide users with personalized services, but customers do not want their privacy to be leaked. In order to solve this problem, some rules have been proposed:

1. If the user's operating system is changed but the IP does not change in the log, it is considered a different user.
2. If there is no hyperlink relationship between the page that the user visits and the page that the user has browsed, it is considered that the two visits come from different users. In order to have a clearer understanding of the process of data preprocessing, the author listed a website in Figure 5. The processed logs are shown in Table 2. Through the analysis of Table 2, it can be seen that there are several records from the same address, but even if the address is the same, it cannot be regarded as a user due to the difference of its agents.

Although these rules cannot be used to identify users with 100% accuracy, these rules can still provide us with some help in identifying users due to the existence of the same set of pages. The user's browsing of the Web is unpredictable. In response to this problem, the concept of conversation is proposed. Conversation refers to the interaction between the user and the server.

In the process of data mining, the choice of technology is not the only one. How to choose the right technology is the most important issue. In terms of analyzing log data, most software is formed on the basis of statistical technology, and very few software is formed on the basis of data mining. Since the twenty-first century, many companies that sell Web and use mining products have disappeared from the market. Currently, a part of the Web uses log analyzers for data mining. Although researchers have proposed many mining techniques, these techniques still cannot be applied to actual data mining, so the research must be strengthened. Main research projects and products for Web usage mining are shown in Table 3.

As time goes by, new data will continue to be generated. Traditional data mining methods can no longer be applied to data mining, and the knowledge that has been mined also needs to be updated. The update process is not to remine the data, but to update the changed part of the data. The incremental mining method must be used when the scale of dynamic data is large. In general, the incremental algorithm only needs a small amount of time, and the calculation is relatively simple. Mining logs is to interact with users. Knowledge must be updated on the basis of existing models. The new knowledge obtained must be classified. In this process, the best plan and optimal parameter values must be selected. The specific process is as follows:

1. Web logs must be processed first, users and sessions must be identified, data must be evolved, and paths must be filled to obtain transaction data.
2. Further processing the data, transforming the data structure is to adapt to the mining algorithm.
3. On the basis of existing knowledge, the data are updated through incremental mining algorithms. Cluster analysis is a method of dividing unsupervised data. It can be applied to analyze spatial data and analyze customer relationships and aspects. In the economic field, after analyzing the consumer's data, the consumer's consumption habits can be grasped, which will help marketers formulate personalized marketing strategies. How to obtain knowledge from Web data is the focus of the current research. Cluster analysis can also be applied in the field of bioinformatics, which can classify animals and plants and determine similar genes in animals. In addition, there are also applications of cluster analysis in spatial geographic data analysis, which can identify land uses. In this article, the author introduces the concepts of clustering and fuzzy sets and proposes a clustering algorithm. When applying this method, the data must be segmented first, a fuzzy graph must be established, and incremental data and uneven density must be established. The data are clustered. Through a lot of practice, it can be known that the clustering algorithm can quickly get the correct result.

### 4.3. Data Structure in Clustering Algorithm

When people observe an entity, they usually give the entity certain attributes. If there are *n* entities in the entity set, then there are *n* vectors to describe the entity.

(14) $$\begin{array}{c} \left[\begin{array}{ccccc} x_{11} & \cdots & x_{1j} & \cdots & x_{1p}\\ \cdots & \cdots & \cdots & \cdots & \cdots \\ x_{i1} & \cdots & x_{ij} & \cdots & x_{ip}\\ \cdots & \cdots & \cdots & \cdots & \cdots \\ x_{n1} & \cdots & x_{nj} & \cdots & x_{np} \end{array}\right]\text{.} \end{array}$$

In order to analyze the characteristics of entities and examine the similarities between entities, a dissimilarity matrix is proposed.

(15) $$\begin{array}{c} \left[\begin{array}{ccccc} 0 & d\left(1,2\right) & d\left(1,3\right) & \cdots & d\left(1,n\right)\\ d\left(2,1\right) & 0 & d\left(2,3\right) & \cdots & d\left(2,n\right)\\ d\left(3,1\right) & d\left(3,2\right) & 0 & \cdots & d\left(3,n\right)\\ \vdots & \vdots & \vdots & \cdots & \vdots \\ d\left(n,n\right) & d\left(n,2\right) & d\left(n,3\right) & \cdots & 0 \end{array}\right]\text{.} \end{array}$$

Normally, the rows and columns of the matrix represent different meanings, but in the dissimilarity matrix, the rows and columns all represent the same entity. Some clustering algorithms will use the similarity matrix, and the specific algorithm is as follows:

(16) $$\begin{array}{c} i=\left(x_{i1},x_{i2},x_{in}\right),j=\left(x_{j1,}x_{j2},x_{jn}\right)\text{.} \end{array}$$

The general distance function is as follows:

(17) $$\begin{array}{c} d_{ij}={\left(\overset{n}{\underset{k=1}{\sum }}{\left|x_{ik}-x_{jk}\right|}^{p}\right)}^{1/p}\left(p>0\right)\text{.} \end{array}$$

If *P* = 1, then

(18) $$\begin{array}{c} d_{ij}=\overset{n}{\underset{k=1}{\sum }}\left|x_{ik}-x_{jk}\right|\text{.} \end{array}$$

If *P* = 2, then

(19) $$\begin{array}{c} d_{ij}={\left(\overset{n}{\underset{k=1}{\sum }}{\left|x_{ik}-x_{jk}\right|}^{2}\right)}^{1/2}\text{.} \end{array}$$

If *p* = 3, then

(20) $$\begin{array}{c} d_{ij}=\max_{1\leq k\leq n}\left|x_{ik}-x_{jk}\right|\text{.} \end{array}$$

We transform the coordinate system:

(21) $$\begin{array}{c} d_{ij}={\left[\left(x_{i}-x_{j}\right)V^{-1}{\left(x_{i}-x_{j}\right)}^{T}\right]}^{1/2}\text{.} \end{array}$$

Marantz distance is as follows:

(22) $$\begin{array}{c} r_{ij}=\overset{m}{\underset{k=1}{\sum }}\left|\frac{x_{ik}-x_{jk}}{x_{ik}+x_{jk}}\right|\text{.} \end{array}$$

The similarity coefficient will decrease as the distance increases.

(23) $$\begin{array}{c} r_{ij}=\frac{\left|\overset{n}{\underset{k=1}{\sum }}x_{ik}x_{jk}\right|}{\sqrt{\overset{n}{\underset{k=1}{\sum }}x_{ik}^{2}\overset{n}{\underset{k=1}{\sum }}x_{jk}^{2}}}\text{.} \end{array}$$

The minimum variance criterion is as follows:

(24) $$\begin{array}{c} J_{z}=\frac{1}{2}\overset{e}{\underset{i=1}{\sum }}n_{i}{\bar{s}}_{i},{\bar{s}}_{i}=\frac{1}{n_{i}^{2}}\underset{X\in D_{i}}{\sum }\underset{X\prime \in D_{i}}{\sum }{\left\|X-X\prime \right\|}^{2}\text{.} \end{array}$$

### 4.4. Dynamic Analysis System Based on Web Log Mining

The various techniques and algorithms discussed above are applied to the log mining to establish a model system, and the results obtained can assist decision makers in making decisions. Web log mining needs to process a large number of files. For updated information, incremental mining must be used to fill in the information.

It can perform data mining after knowledge update and can perform incremental processing on data in different modules of the system and add the new knowledge mined to the database in time. The specific functions are as follows:

1. Preprocess one log file or multiple log files to obtain a transaction set or URL and can also process incremental files
2. Perform cluster analysis on the set of things, which can handle user clustering and page clustering
3. After training the neural network, a classification model can be obtained, which can be used to predict the classification of new users
4. The relationship between URL pages can be obtained by analyzing the frequent visits of users

Taking into account the problem of too many pages and too large dimensions, an attribute-oriented induction method is introduced in the preprocessing. This method can reduce the calculation time and improve the efficiency of the algorithm. When using this method, relevant data must be collected first, the attributes of the data must be summarized, and the values must be accumulated. After generalization, knowledge that could not be found before can be found. In order that the data mining after generalization can represent the knowledge of the original things, the hierarchical structure of the original page must be retained when generalization is performed. In generalization, you can remove duplicate pages and simply increase the browsing time. After such processing, the things obtained are called generalized things.

## 5. Conclusion

Genetic algorithm is a random global optimization algorithm that can simulate biological evolution and genetic processes. It can be applied in many fields, such as scientific research and engineering technology. However, there are often some problems when using genetic algorithms. For example, the local search ability is not strong, it cannot reflect the regulation of genetic information on organisms, and it is easy to converge prematurely. These problems make the genetic algorithm unable to reach an accurate optimal solution. In recent years, with the further advancement of research, people have discovered that DNA intelligent systems can reflect the genetic information of birth objects, which is conducive to the development of more powerful and more complex intelligent methods for solving problems. The use of base coding can simulate the genetic mechanism of organisms, promote the genetic algorithm to further simulate the genetic mechanism and gene regulation mechanism of organisms, improve the function of genetic algorithm, increase the accuracy of the algorithm, and find the optimal solution. The research on this algorithm has received extensive attention from domestic and foreign researchers. With the advancement of Internet technology and the rapid growth of data, artificial intelligence has gradually developed. It is an inevitable trend to use artificial intelligence to process data in the era of big data. After analyzing the fuzzy theory, a hierarchical clustering algorithm is proposed, which divides large-scale data, establishes a fuzzy graph, and obtains the clustering result after taking a screenshot of the fuzzy graph. FHC algorithm can effectively cluster clusters of arbitrary shape. And, compared this method with other algorithms, it has advantages in both the clustering quality and running time, and it is a fast and efficient clustering method. The author proposes a system framework based on the incremental analysis system of log mining and implements a prototype system. Log files can be mined, classified, and preprocessed in the prototype system. For incremental data obtained by preprocessing, incremental data mining can be carried out, classification models can be established, and databases can be updated to provide users with personalized services. The author also analyzed the physical fitness of college students and found that most of the students have poor physical fitness and did not develop the habit of daily physical exercise. When researching physical education, it was found that the goals of physical education were vague, the teaching thoughts were backward, and the sports talents were insufficient. A series of problems such as outdated sports assessment standards and insufficient sports equipment are in urgent need of improvement.

## Figures and Tables

**Table 1:** Server log file example.

Record number	Logging
1	220.189.42.159 - - [17/Sep/2006 : 00 : 00 : 01 + 0800] ”GET/blxcy/count show^sp HTTP/1.1″ 200 266
2	220.189.42.159 - ・ [17/Sep/2006 : 00 : 00 : 02 + 0800] ”GET/imagcs/dd 03.jpg HTTP/1.1″ 200 859
3	202.160.178.107 - - [17/Sep/2006 : 00 : 00 : 03 + 0800] ”GET/news print.php?id-28854&catg code = news-01-04 HTTP/1.0″ 200 5229
4	218.71.141.2 • - [17/Sep/2006 : 00 : 00 : 07 + 0800] ”GET/images new/index 171.jpg HTTP/1.1′, 404 1468
5	202.10822.142 - - [17/Sq>/2006 : 00 : 00 : 07 + 0800) ”HEAD/xgxwJist.php?Nid = 31200&page = 16 HTTP/1.1″ 200 379
6	218.71.141.2 - - (17/Sep/2006 : 00 : 00 : 07 + 0800] ”GET/pic/bl_l.gif HTTP/1.1″ 304 266
7	220.189.42.159 ・ • [17/Sep/2006 : 00 : 00 : 14 + 0800] ”GET/pop/051220 2.gif HTTP/1.1″ 200 92688
8	202.108.22.142 - - [17/Sep/2006 : 00 : 00 : 16 + 0800] ”HEAD/xgxw list.php?Nid = 28404&page = 934 HTTP/1.1″ 200 379
9	202.108.22.142 - - [17/Sep/2006 : 00 : 00 : 26 + 0800] ”HEAD/new$_print.php?id = 29842&catg_code = news-03-18-4 HTTP/1.1″ 200 379

**Table 2:** Log after data purification.

Record number	Logging
1		220.189.42.159 - - [17/Sep/2006 : 00 : 00 : 01 + 0800] ”GET/blxcy/count show^sp HTTP/1.1″ 200 266				
2		220.189.42.159 - ・ [17/Sep/2006 : 00 : 00 : 02 + 0800] ”GET/imagcs/dd 03.jpg HTTP/1.1″ 200 859				
3		202.160.178.107 - - [17/Sep/2006 : 00 : 00 : 03 + 0800] ”GET/news print.php?id-28854&catg code = news-01-04 HTTP/1.0″ 200 5229				
4		218.71.141.2 • - [17/Sep/2006 : 00 : 00 : 07 + 0800] ”GET/images new/index 171.jpg HTTP/1.1′, 404 1468				
5		202.10822.142 - - [17/Sq>/2006 : 00 : 00 : 07 + 0800) ”HEAD/xgxwJist.php?Nid = 31200&page = 16 HTTP/1.1″ 200 379				
6		218.71.141.2 - - (17/Sep/2006 : 00 : 00 : 07 + 0800] ”GET/pic/bl_l.gif HTTP/1.1″ 304 266				
7		220.189.42.159 ・ • [17/Sep/2006 : 00 : 00 : 14 + 0800] ”GET/pop/051220 2.gif HTTP/1.1″ 200 92688				
8		202.108.22.142 - - [17/Sep/2006 : 00 : 00 : 16 + 0800] ”HEAD/xgxw list.php?Nid = 28404&page = 934 HTTP/1.1″ 200 379				
9		202.108.22.142 - - [17/Sep/2006 : 00 : 00 : 26 + 0800] ”HEAD/new$_print.php?id = 29842&catg_code = news-03-18-4 HTTP/1.1″ 200 379				
10	218.0.12 4.90	2007-01-22 05 : 57 : 35	GET P3.html	P13.hlm 1	Mozilia/4.0+(compatible;+ MSlE+6.0b;+Windows NT5.0)
11	218.0.12 4.90	2007-01-22 05 : 57 : 53	GET P13.html	P3.html	Mozilla/4.0+(compatible; +MSlE+5.5;+Windows+98)
12	2U.0.12 4.90	2007-01-22 05 : 58 : 07	GET P31.hlml	P3.html	Mozilla/4.0+(compatib!e;+ MSIE+6.0b;+Windows NT5.0)
13	218.0.12 4.90	20070–22 05 : 59 : 44	GET P32.html	P3.html	Mozilla/4.0+(compaiiblcr∗- MSIE%.0b;+Windows NT5.0)
14	218.0.12 4.90	2007-01-22 07 : 15 : 28	GET Plhtml	M.html	Mozilia/4.(H(compatible;+ MSIE+^.0b;+Windows NT5.0)
15	218.0.12 4.90	2007-01-22 07 : 17X)5	GET PI3.html	P3.html	Mozilla/4.0+(compatiblc;+ MSIE+6.0b;+Windowi NT5.0)

**Table 3:** Main research projects and products for Web usage mining.

Use	Technology	Data source
Personalized design	Clustering, association rules, and relational Markov model	Web server
Personalized design	Clustering	Web server
Personalized design	—	Client
Personalized design	Clustering, classification	Web server
Personalized design	Clustering	Web server
Nonsexual design	Association rules and clustering	Web server
Personalized design	—	Web server
Personalized design	Clustering and sequence mode	Web server
Prefetching and buffering	Classification, association rules, and sequence patterns	Proxy server
Prefetching and buffering	Markov model	Web server
Prefetching and buffering	Sequence mode	Web server
Prefetching and buffering	Association rules	Proxy server
Prefetching and buffering	Markov model	Web server
Prefetching and buffering	Union rules	Web server
Design	Classification and sequence mode	Web server
Design	Multimedia synchronization	Web server
Design	Binary code	Web server
Design	Markov model	Web server
E-commerce	Classification, association rules, and sequence patterns	Web server
E-commerce	Clustering	Web server
E-commerce	Fuzzy logic, clustering, and genetic algorithm	Web server

## Data Availability

The data used to support the findings of this study are available from the author upon request.
